# An easy-to-use function to assess deep space radiation in human brains

**DOI:** 10.1038/s41598-021-90695-5

**Published:** 2021-06-03

**Authors:** Salman Khaksarighiri, Jingnan Guo, Robert Wimmer-Schweingruber, Livio Narici

**Affiliations:** 1 Institute of Experimental and Applied Physics, University of Kiel, 24118 Kiel, Germany; 2 School of Earth and Space Sciences, University of Science and Technology of China, Hefei, 230026 China; 3 CAS Center for Excellence in Comparative Planetology, USTC, Hefei, China; 4 Departments of Physics, The University of Rome ’Tor Vergata’, Rome, Italy; 5 INFN Roma Tor Vergata, Rome, Italy

**Keywords:** Astrobiology, Space physics, Risk factors

## Abstract

Health risks from radiation exposure in space are an important factor for astronauts’ safety as they venture on long-duration missions to the Moon or Mars. It is important to assess the radiation level inside the human brain to evaluate the possible hazardous effects on the central nervous system especially during solar energetic particle (SEP) events. We use a realistic model of the head/brain structure and calculate the radiation deposit therein by realistic SEP events, also under various shielding scenarios. We then determine the relation between the radiation dose deposited in different parts of the brain and the properties of the SEP events and obtain some simple and ready-to-use functions which can be used to quickly and reliably forecast the event dose in the brain. Such a novel tool can be used from fast nowcasting of the consequences of SEP events to optimization of shielding systems and other mitigation strategies of astronauts in space.

## Introduction

Among various issues that may cause health problems for astronauts, the hazard from radiation on interplanetary flights is currently one of the major obstacles, especially for long-duration missions to outer space, e.g., a manned mission to Mars^1,2^. While space radiation research has grown swiftly in recent years, substantial uncertainties remain in predicting and extrapolating the responses of humans to radiation exposure. One of the reasons may be because only 24 human beings have ventured beyond the protective envelope of the Earth’s magnetosphere for a maximum of approximately 12 days (Apollo 17)^3^.

Exposure to charged particles during long-term missions in space, where high-energy particles are more abundant than in Earth orbit, can lead to substantial biomedical or health risks for astronauts^3,4^. Space radiation risks include carcinogenesis, degenerative tissue effects, central nervous system (CNS) decrements^5,6^, heart diseases^7,8^ and acute radiation syndrome^9–12^. Acute effects are possible when astronauts are exposed to large SEP events, which produce a high radiation dose^13,14^. On the other hand, a major concern of space radiation is the long-term effect on astronauts, which can include cataracts, an increased chance of cancer, and some health effects are even thought to be passed on to the next generations by mutated genes^1,5,9,15^.

### Radiation environment in deep space

The deep-space radiation environment is determined by two major sources of ionizing radiation, with Galactic Cosmic Rays (GCRs) and Solar Energetic Particle (SEP) events. Protons and helium ions comprise about $87\%$ and $12\%$ of GCRs, respectively^16^, with additional contributions by heavier nuclei such as carbon, nitrogen, oxygen, and iron. Despite their small abundance of about 1%, these heavy ions are of particular concern for manned space missions because of their relatively high Linear Energy Transfer, and the uncertainties of their biological effects. Once they have entered the solar system, the GCRs are modulated by the heliospheric magnetic field, which changes with the 11-year solar activity cycle [e.g.^17^]. The flux of GCR protons may be two orders of magnitude higher during solar minimum than during solar maximum years at energies below about 100 MeV as shown in Fig. 2b (as obtained from a GCR model that will be explained in detail in “Calculation method and setups”). The flux of GCRs is low, but their influence integrated over a typical duration of a Mars mission of 3 years can be substantial. A risk assessment study of exposure to space travelers^18^ estimates that 20 million out of 43 million hippocampus cell nuclei will be directly hit by one or more particles, with charge Z >15 assuming the 1972 GCR spectrum after the peak year of solar cycle 20.

On the other hand, SEP events are dominated by protons and electrons, which are accelerated in sporadic solar eruptions such as flares or shocks driven by Coronal Mass Ejection (CME) (e.g.^19^). Small and flare-associated SEP events can occur at any time during the solar cycle, whereas larger SEP events tend to occur during periods of high solar activity. They can lead to high radiation doses in short time intervals^20.^

### Radiation effects on human brains

**Figure 1:**
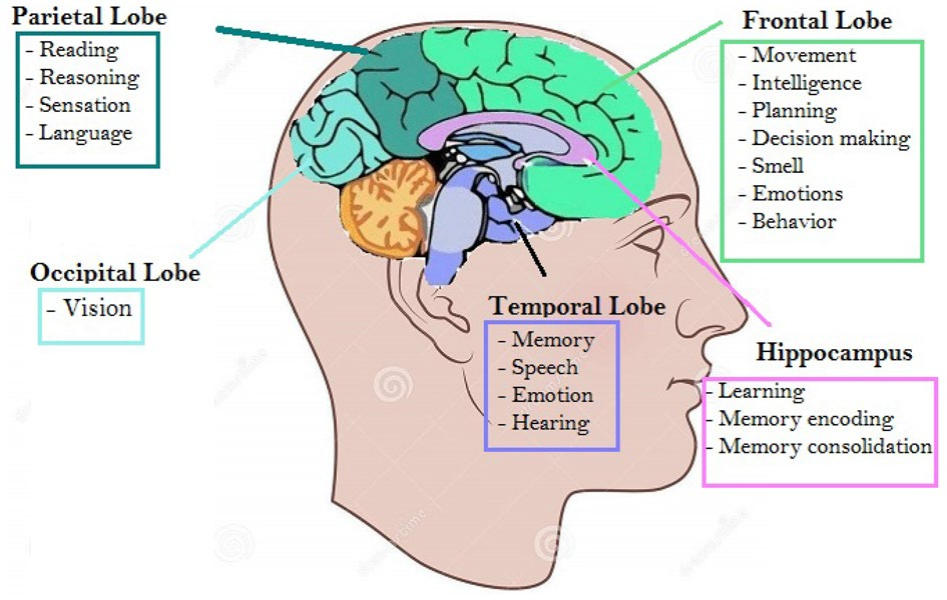
Structures of a human brain including different lobes and hippocampus with their corresponding major functions adapted from^27^. The position might appear different due to the selected 2D cut of the 3D structure. More descriptions can be found in “Radiation effects on human brains”.

Our brain is the command center for the human nervous system, which interprets information from the outside world and directs our body’s internal functions. The cerebrum is the largest portion of the brain containing the cerebral cortex, which is responsible for most of the actual information processing in the brain. The cerebral cortex is divided into four cortex lobes: the frontal lobe, temporal lobe, parietal lobe, and occipital lobe. These lobes are responsible for various functions in the body that include everything from sensory perception to decision-making and problem-solving^21^, as indicated in Fig. 1. We also consider the hippocampus region in our study, which is a structure within the medial aspect of the temporal lobe that can be identified as a layer of densely packed neurons and plays a vital role in regulating learning, memory encoding, memory consolidation, and spatial navigation^22^.

The brain ventricular is a highly conserved aspect of brain structure, a series of connected cavities lying deep within the brain, filled with cerebrospinal fluid (CSF). These structures are responsible for the production, transport, and removal of CSF. The brain ventricles can enlarge during spaceflight^23–26^. While this is a real change in the structure of the brain, its relevance in these studies is uncertain, and there is no model for it under zero gravity conditions. Therefore, we did not include this effect in our simulations of a realistic brain structure.

Different radiation effects have been reported from patients experiencing conventional cranium radiotherapy or treatments for glioma brain tumor such as, adverse effects and behavioral changes in CNS, long-term anxiety, depression, disturbances in learning, memory, processing speed, attention, and cognitive flexibility^28–32^. Acute or short-term radiation effects may occur during or immediately after the course of radiation, such as fatigue or hair loss. Late or long-term effects include white matter changes, radionecrosis, neuropsychological and endocrine changes (hormonal)^32–35^.

Animal models and experimental studies of exposing mice to doses of radiation corresponding to that of a cruise to Mars^36^ suggest that the radiation exposure may impair cellular signaling in the hippocampus, a brain region bound to learning and memory^37^, and may also cause damage in short-term memory as well as disturb the crew’s performance and decision making procedure^38^. It also reveals the capability of radiation to significantly decrease the structural complexity and synaptic integrity of neurons throughout different regions of the brain, inducing compromised cognitive performance of mice^29^.

To better assess the space radiation effect on the brain, we study the GCRs and SEP events induced dose deposit in different lobes and in the hippocampus region of the brain by calculating the dose distribution inside an actual three-dimensional head structure extracted from computed tomography (CT) images. Thus this paper consists of three parts. First, we introduce and describe the method for calculating radiation doses inside the brain. Second, we estimate the GCR radiation dose deposit inside different lobes and in the hippocampus region of the brain, with various depths of shielding around the head. Third, we consider the consequence of SEP events using SEP spectral data of 50 historical events and find some easy-to-use functions, which can quickly convert the SEP event intensity at a certain pivot energy to the final dose deposit in the brain, following a method developed by^39^. Finally, we evaluate the implications of our calculations in the context of astronauts performing interplanetary space missions.

One important output of this work is to provide a quantitative assessment that could help to produce a possible ‘alarm’ for the SEPs. Our results would be used to predict the importance of the coming radiation event and, together with the knowledge from radiation treatments for cancer, and the growing understanding of the influence of radiation on cognitive functions^40^, to forecast the level of radiation effects and the subsequent risks, providing grounds for the related alarm.

## Calculation method and setups

Previous studies of radiation effects on CNS have most commonly reported measurements or calculations of Dose (Gy) to characterize the radiation risk. Alternatively, in some works, it has been suggested that particle fluence or the measure of the number of electrons emitted per unit track length are better predictors for the effectiveness of different particles for neurobehavioral dysfunction^40^. In the current study, we stay with the basic physical unit of the radiation dose, i.e., energy deposit per unit mass, as the results can be more easily related to the biological radiation experiments.

In a previous study^41^, we calculated the dose distribution in different parts of the human brain using actual head densities extracted from CT images. The particle transport simulations have been carried out using version 10.4 of Geant4 toolkit [GEometry And Tracking^42,43^], a well-established and three-dimensional Monte Carlo particle transport tool.

We used a matrix approach following^44,45^ to obtain “brain response functions” (BRFs) of the dose dependence on the primary particle type and energy. A BRF is a probabilistic description of the deposited energy of a primary particle, with a defined type and energy, in the brain. All possible interactions, which may be triggered when the primary particle and its secondaries penetrate through the head, are included in the BRF. Primary particles of protons and helium ions, as well as carbon, nitrogen, and iron, are considered to construct these BRFs^41^. The BRF has a unit of dose per fluence of primary particles and can be used to fold (i.e., multiply per energy bin and then integrate) with a given primary particle spectrum for obtaining the radiation dose inside the brain, similar to the approach in^46^.

*Extra shield around the head*: As humans in space would always have a certain kind of shielding protection, we simulated various depth of aluminum shielding (0.2 cm, 0.5 cm, 2 cm, 5 cm, and 10 cm) between the radiation source and the brain. Aluminum(Al) was used because it is a conventional choice used in the community, so that our result can be more easily compared to previous works^47,48^. The thickness chosen here can be approximating a helmet, spacesuit, and other possible shielding depths of a habitat^41^. It is important to note that primary GCRs and SEP events passing through shielding materials may undergo inelastic interactions with the ambient atomic nuclei losing some or all their energy and also creating secondary particles via spallation and fragmentation processes, resulting in a radiation field including both primary and secondary particle radiation inside the space vehicle (e.g.^49^). These secondaries including neutrons which are highly relevant for their biological effects are modeled and propagated further in our models and they may also contribute to the dose in the brain within the shielding.

*GCRs in deep space:* In “Deep-space radiation from GCRs” we fold the BRFs with deep space GCR spectra under different solar modulations as predicted by the Badhwar O’Neil model^50^ and shown in Fig. 2b. We show results for solar modulation potentials ($\Phi$) between 300 MV and 1200 MV. A large $\Phi$ value corresponds to a strong solar modulation with enhanced interplanetary magnetic fields that depress the GCR flux around the solar maximum; a smaller $\Phi$ instead represents weaker solar activities with less-modulated and thus higher GCR flux. Dose deposits in different lobes of the brain and in the hippocampus region are then calculated based on various solar modulation conditions and different Al-shielding depths.

*SEP events in deep space:* In the second part of this work, we use BRFs to fold with a variety of SEP events shown in Fig. 2b. The SEP spectra represent some historical large events from August 1997 to 2006, reconstructed from measurements of the Geostationary Operational Environmental Satellite (GOES) and the Advanced Composition Explorer (ACE) satellite at the deep space environment near Earth^51^. Such events show different spectral properties (intensities and shapes), which complicate the assessment of the radiation dose experienced by astronauts. We, therefore, carry out a statistical study of the correlation between the dose deposit in the brain by each event and the SEP spectral parameters and obtain some easy-to-use functions. These functions rely on the so-called pivot energy of the original SEP spectra at which the spectral index does not influence the final dose deposit^39^.

## Simulation results and discussion

**Figure 2:**
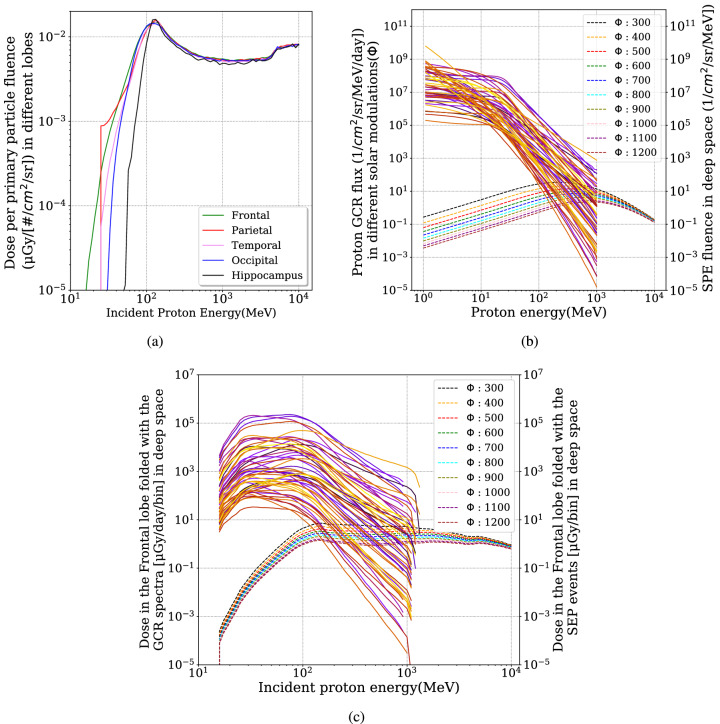
Folding the Brain Response Functions (BRFs) with primary GCR/SEP protons to calculate the dose rate inside different lobes, and in the hippocampus region of the brain as described in “Application of the simulated results—the procedure to apply our ready functions for calculating the resulted radiation inside a brain”. (**a**) BRFs as calculated in^41^ for protons. (**b**) Energy- and time-differential flux of GCRs under different solar modulation potential (MV) (dashed curves) and the energy-differential but event time-integrated fluence of SEP events (solid lines) in deep space. (**c**) Convolutions of the GCR/SEP particle spectra of panel (**b**) with the BRF for the frontal lobe.

### Application of the simulated results—the procedure to apply our ready functions for calculating the resulted radiation inside a brain

In this section, we demonstrate how to apply readily-calculated BRFs to obtain the radiation dose rate inside the four main lobes, and in the hippocampus region of an astronaut’s brain. The procedure of the application and the results for deep-space exposure to GCRs are shown in Fig. 2.

The BRFs for protons in the different lobes, and in the hippocampus region of the brain are plotted in Fig. 2a. After folding them with the various possible input spectra, shown in panel (b) of Fig. 2, i.e., multiplying the values of BRF with the spectra flux in each corresponding energy-bin, one obtains the dose rate in the different lobes as a function of energy as shown in panel (c). The integration of the total dose rate over all energies gives the radiation deposit by the primary GCR protons in the brain (or at a certain part of the brain). Mathematically, the procedure described above can be summarized as given in Eq. (1).

1 $$\begin{aligned} \dot{D} =\left\{ \frac{D}{f}\right\} \times F , \end{aligned}$$

where *D*(µGy) is the dose deposit in each lobe resulted from simulation, *f* is the fluence of the source used in the simulation in units of particles $\hbox {cm}^{-2}$ $\hbox {sr}^{-1}$ $\hbox {MeV}^{-1}$, *F* represents the double-differential (both energy- and time-differential) flux of GCRs in units of particles $\hbox {cm}^{-2}$ $\hbox {sr}^{-1}$ $\hbox {MeV}^{-1}$ $\hbox {s}^{-1}$, finally, $\dot{D}$ is the GCR-induced dose rate in each lobe given in µGy/s. We note that when applying the above method to SEP events, *F* is integrated over the respective time duration for each event so that the obtained dose is not time-differential and has the unit of µGy.

### Deep-space radiation from GCRs

**Figure 3:**
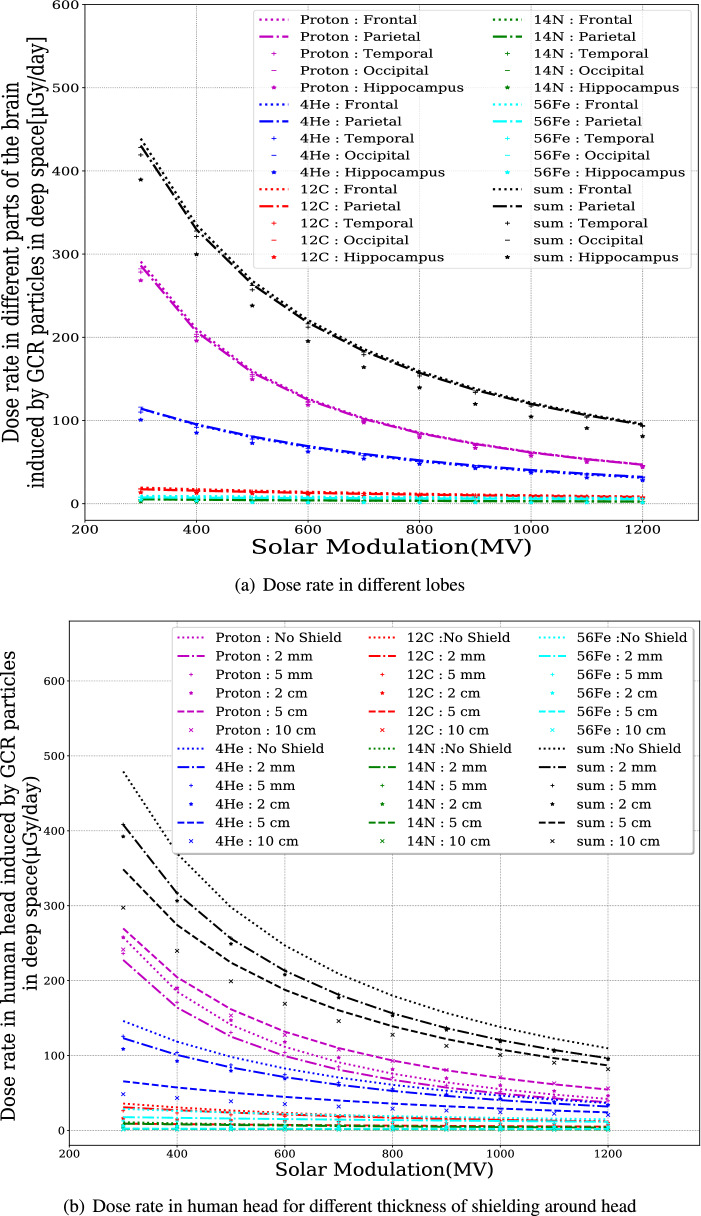
(**a**) The dose rates in different parts of the brain induced by GCR particles in deep space without shielding protection. (**b**) The dose rates in the human head under different thickness of shielding. The primary source GCRs include protons, helium and heavier ions such as carbon, nitrogen and iron under different solar modulation conditions. The summed dose rates from all these primary particles under different conditions (i.e., solar modulations, shielding depth and different lobes) are shown in black.

We used the procedure discussed in “Application of the simulated results—the procedure to apply our ready functions for calculating the resulted radiation inside a brain” to calculate the dose rate in different parts of the brain for a range of GCR spectra at different solar activities throughout the solar cycle. The GCR spectra are obtained based on the Badwhar O’Neil model^50^, which depends on the parameter solar modulation potential, $\Phi$. We made the calculations using different $\Phi$ values ranging from 300 MV (solar minimum) to 1200 MV (solar maximum). The results are shown in Fig. 3a for dose rate in different lobes without shielding. As expected, the differences between the dose rates in the different lobes are hardly discernible, but is smaller in the hippocampus region. This difference is mainly due to the contribution of low energy particles which can be more easily stopped before reaching hippocampus, which is located in the inner region of the temporal lobe and more shielded compared to different lobes. The dose rates, that concern all modeled GCR particles in deep space, vary between about 100 and 440 µGy/day for solar activity maximum and minimum. However, we note that our estimation of the GCR-induced dose rate might be slightly smaller than the expected values as we have cut off the particles above 10 GeV in our simulations since particle flux above this energy decreases versus the energy following a power-law.

Figure 3b shows the dose rate for the entire human head given different solar modulation potentials, $\Phi$, for different particle species considered here, and for different shielding, as explained in “Calculation method and setups”. Some subtle differences can be seen. For instance, the highest dose rate from protons is not for the unshielded scenario as for He-nuclei, but for a shielding thickness of 5 cm Al-equivalent. Nevertheless, the total dose rate from all primary GCR particle species shows a decrease when the shielding is increased. At solar minimum, a shielding of 2 mm of Al results in an overall reduction by approximately 14%. 27% reduction is reached with shielding by 5 cm of Al. A further increase in shielding thickness to 10 cm of Al results in a total reduction of 37%. These values are reduced somewhat during solar maximum conditions (12%, 20%, and 25%, respectively).

Exposure to 0.5 Gy of simulated GCR radiation, containing a mixture of different particle species may result in some long-term deficits in the recognition memory system of male mice^52^. Approximating this as an “upper limit” of the accumulated GCR radiation for brains in space despite of it being different from those of the mice, we can derive the appropriate shielding depth and the allowed duration of the space mission under different solar modulation conditions. In particular, during solar minimum when the GCR flux is around its maximum value, with 2 mm of shielding, the GCR radiation in the brain can be reduced to about 400 µGy/day. On the other hand, during solar maximum, the GCR radiation in brains is already much lower, and extra shielding is not that necessary, but surely helpful.

### Deep-space radiation from SEP events

Alternatively, the short-term sporadic solar energetic particles events generated during solar eruptions may result in some sudden and drastic enhancement of the radiation in the brains of astronauts in space. To assess the potential radiation effects associated with these SEP events, we evaluate the effect of 53 large SEP events^51^ without and with the various shielding thicknesses used in the previous section “Deep-space radiation from GCRs”. Table 1 lists dose deposits in the human head for 35 of these 53 SEP events. They were chosen as they deposit more than 10 cGy dose in the human head without shielding.

In the case of no shielding around the head, the dose in different lobes of the human brain is considerably less than the dose averaged in the entire human head because of the human cranium with an average of 6.5 mm thickness^53^ protects the brain against low-energy ions. Moreover, the dose in the frontal lobe is slightly higher than other lobes, and the hippocampus experienced the lowest dose among the studied regions of the brain in our work. This is the same effect as already observed for low energy GCRs (below 100 MeV/nuc). The reason is that the frontal lobe has a larger portion at the outer edge of the brain and is more exposed to the radiation contribution from low-energy primary particles, but the hippocampus is located deep in the temporal lobe, and it is the most shielded part compared to the others. This effect is more obvious for SEP events because they have a larger contribution from low energy particles in comparison to GCRs.

The results reported in this table clearly show that even a modest shielding of only 2 mm of Al can reduce the dose in the head by more than 50% in the case of SEP events. Previous studies considered the Space-Permissible Exposure Limit (SPEL) for short-term radiation exposure of CNS to be about 50 cGy in 30-days^54^. We mark the event doses that exceed 50 cGy  in bold font in Table 1. As shown, even under 2 cm of Al shielding, some events exceed the 30-days SPEL in the human head. Besides, the enhanced solar activity during the maximum of a solar cycle often leads to multiple SEP events taking place within the course of days or weeks such as the few events associated with the famous Halloween storm in 2003 as shown in Table 1.  It is therefore important to forecast SEP events and prepare with extra shielding during hazardous situations like this.

**Table 1:** Resulting dose (cGy) from 35 SEP events in a human head considering different thicknesses of Al as a shield around an astronaut’s head/body. Dose deposits in different lobes of the human brain protected by 2 mm of shield are also shown. Those event doses that exceed 50 cGy are shown in bold font. These 35 events are chosen from the list as they would deposit more than 10 cGy of dose in the head without shielding (second column).

	Dose(cGy) in a human head shielded by different thicknesses of Al						Dose(cGy) in different lobes of a human brain				
Events	Without shield	2 mm	5 mm	2 cm	5 cm	10 cm	Frontal	Parietal	Temporal	Occipital	Hippocampus
	(cGy)	Al (cGy)	Al (cGy)	Al (cGy)	Al (cGy)	Al (cGy)	(cGy)	(cGy)	(cGy)	(cGy)	(cGy)
6 Nov 1997	**52**	12	4	0.9	0.2	0	6	4.6	4.4	4.3	1.5
20 Apr 1998	**123**	39	13	2	0.4	0.1	13	9	9	8	5
24 Aug 1998	32	4	1.4	0.2	0	0	0.63	0.5	0.4	0.4	0.17
30 Sep 1998	31	5	2	0.4	0	0	1	0.8	0.7	0.7	0.3
14 Nov 1998	13	4	2	0	0	0	1.4	1.17	1.14	1.2	0.6
14 July 2000	**2005**	**741**	**345**	**78**	22	6	179	144	139	141	62
8 Nov 2000	**1354**	**542**	**283**	**71**	20	5	159	132	128	130	64
26 Nov 2000	26	4	1.5	0.3	0	0	0.69	0.53	0.5	0.5	0.19
2 Feb 2001	**58**	15	6	1.1	0.3	0	2.9	2.3	2.1	2.1	0.85
10 Feb 2001	10	2	0.7	0.1	0	0	0.36	0.28	0.26	0.25	0.1
12 Feb 2001	19	1	0.2	0	0	0	0.07	0.05	0.05	0.04	0
15 Feb 2001	**79**	39	28	14	7	3	21	19	18	17	12
18 Feb 2001	20	9	6	3	1.4	0.6	4.6	4.1	4.1	4	2.6
16 Aug 2001	28	13	8	3	1.4	0.6	5.7	4.8	4.8	4.3	2.8
24 Sep 2001	**426**	**92**	41	9	2	0.8	21	17	16	16	7
1 Oct 2001	**70**	8	1.7	0.1	0	0	0.6	0.4	0.35	0.3	0.07
4 Nov 2001	**2255**	**736**	**256**	34	7	1.7	110	82	77	76	26
22 Nov 2001	**591**	**83**	24	2	0.5	0.1	9	7	7	6	2
26 Dec 2001	48	18	10	3	1.3	0.5	6.3	5.7	5.3	5.5	2.9
21 Apr 2002	**267**	**92**	36	6	1.4	0.4	16.6	12.9	12.2	12	4.6
24 Aug 2002	29	9	4	1.3	0.5	0.2	2.8	2.5	2.3	2.3	1
26 Oct 2003	12	3	1.6	0.4	0.1	0	0.9	0.7	0.7	0.7	0.3
28 Oct 2003	**1268**	**357**	**174**	43	13	4	94	77	76	78	35
29 Oct 2003	**244**	**68**	36	10	3	1.2	21	18	17	18	9
2 Nov 2003	**89**	34	15	3	1	0.3	8	7	7	6	3
7 Nov 2004	12	1.5	0.5	0	0	0	0.27	0.21	0.2	0.2	0.07
10 Nov 2004	12	6	4	2	1.4	0.3	3.7	3.4	3.3	3.5	2.3
16 Jan 2005	27	4	2	0.4	0.1	0	1	0.78	0.74	0.7	0.3
17 Jan 2005	**362**	**108**	35	4	0.9	0.2	15	11	11	10	3
20 Jan 2005	**158**	**90**	**69**	37	20	10	53	49	48	50	34
13 May 2005	22	1.3	0.38	0	0	0	0.16	0.11	0.1	0.1	0.03
22 Aug 2005	19	2	0.6	0	0	0	0.24	0.18	0.16	0.15	0.05
7 Sep 2005	**135**	25	10	1.7	0.4	0.1	4.6	3.6	3.4	3	1.3
6 Dec 2006	**174**	44	21	5	1.5	0.4	11	8.9	8.6	8.4	3.9
13 Dec 2006	**81**	40	26	10	4	1.6	17	15	14.7	14.5	8.5

**Figure 4:**
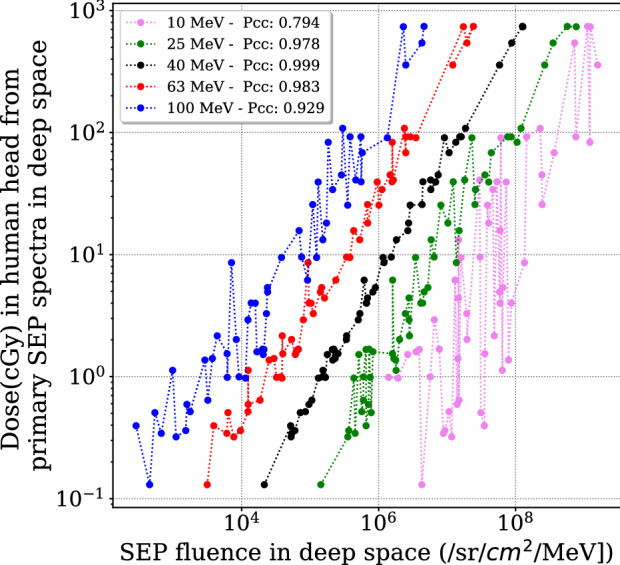
Dose (cGy) deposit in the human head, with 2 mm of shielding around, induced by each SEP event under study versus the original SEP fluence at a few specific energies as marked in different colors and labeled in the legend with the Pearson correlation coefficients (Pcc) for different cases.

A statistical study of SEP-induced Martian surface radiation by events with different properties such as their energy range, intensity, and the power-law index has been performed by^39^. They found a good correlation between the induced radiation on Mars and the fluence of the initial SEP spectra. In particular, they discovered a pivot energy ($\sim$ 300 MeV) at which the SEP flux alone can be used to determine the Martian surface dose rate for large SEP events. Based on the same idea, we try to correlate our calculated dose deposit in the human head under different scenarios (shielding and different lobes) versus the original SEP event fluence at a few specific energies. As shown in Fig. 4, the dose deposit in the head is nicely correlated with the original SEP event fluence at selected energies, in particular at 40 MeV, where the Pearson correlation coefficient is nearly unity. This means that with a given fluence at this pivot energy, other information of the SEP spectra does not effectively influence the radiation deposit in the brain. This feature can be related to the balance of dose contribution by SEP events below and above the pivot energy^39^. A SEP spectrum is often described as a power-law distribution. When the slope of the power-law changes from e.g., − 4 to − 2, the reduction of the dose contribution from particles below the pivot energy is compensated by the increased dose from particles above this point.

Here we obtain the pivot point being 40 MeV in case of dose deposit in the head with a 2 mm Al shield. This pivot energy is lower than the case of Mars because of the thinner shielding considered compared to the Martian atmosphere. For a thicker shielding, we expect this pivot point to shift towards a higher energy. We also fit the correlation of dose deposit [cGy] versus the SEP events fluence [particles $\hbox {sr}^{-1}$ $\hbox {cm}^{-2}$ $\hbox {MeV}^{-1}$] at the pivot energy, for different events, with a linear function as shown in Fig. 4 and Eq. (2) as follows :

2 $$\begin{aligned} D_{\mathrm{Brain}} = a \cdot I_{\mathrm{{Pivot~Energy}}} + b \end{aligned}$$

where *a* has the unit of cGy sr $\hbox {cm}^{2}$ MeV and *b* with unit of cGy. Their fitted values are represented in table 2 for different scenarios. The obtained function can be used to directly derive the dose deposit in the head (or different parts of the brain) using the SEP events fluence at the single energy and thus can significantly simplify the radiation forecast for future manned missions in space upon the onset of SEP events.

**Figure 5:**
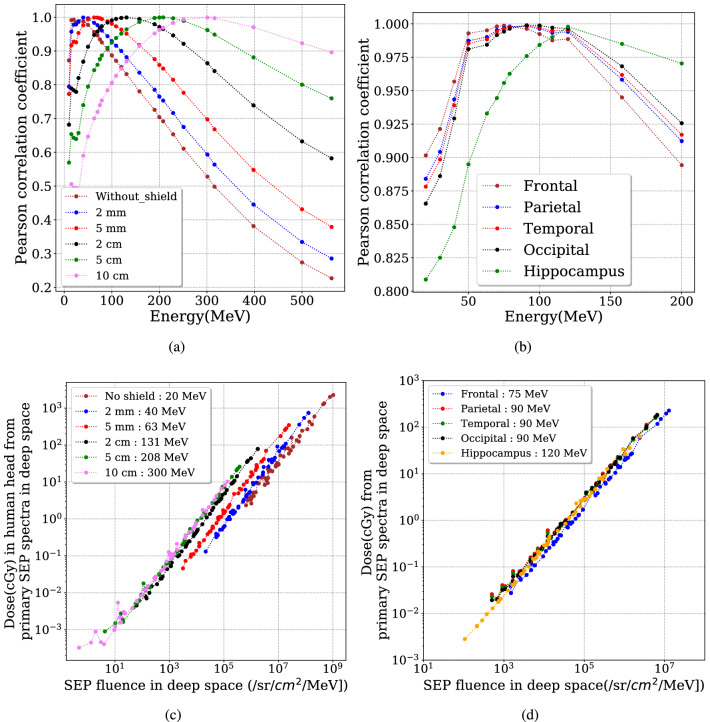
Upper panels: correlation coefficients between the event dose and SEP fluence of each event at different energies for (**a**) various thicknesses of Al around a human head and (**b**) different lobes of the human brain with 2 mm of Al shielding. Lower panels: The dose (cGy) deposit in a human head from primary SEP spectra in deep space versus SEP events fluence at the determined pivot energy for (**c**) a human head with different Al thickness and (**d**) different lobes of the brain under a 2 mm of Al shield.

To better locate the pivot energy of the dose deposit in the brain under different cases studied, we calculate the Pearson correlation coefficient (Pcc) of the obtained dose versus the SEP fluence of each event at different energies. In Fig. 5a, we show the Pcc values versus energies of the original SEP spectra for various cases of shielding around the head. As expected, the best Pcc for each case has the peak value around 1 at a certain energy that is the pivot energy to be found. The pivot energy for a thicker shield shifts towards a larger value and equals about 300 MeV for 10 cm of Al shield. This is the same pivot energy found by^39^ as the accumulated shielding depth in this case (27 g/$\hbox {cm}^2$) is comparable to the Martian atmospheric depth studied by ^41^ (the vertical column mass is about 22 g/$\hbox {cm}^2$ of $\hbox {CO}_2$ and the average shielding depth is slightly larger). In Fig. 5b, the pivot energy is determined, for dose deposit in four lobes under 2 mm of Al shielding, to be 75 MeV for the frontal lobe and 90 MeV for the other three lobes and increases to 120 MeV for the hippocampus region as this part is embedded deep into temporal lobe and more shielded compared to other lobes. On the other hand, the slightly smaller pivot energy for the frontal lobe also reveals the fact that it is the least shielded lobe compared to the others. Fig. 5c,d plot the correlation of the dose deposit versus the SEP fluence at the determined pivot energy, which is obtained with the best Pcc values larger than 0.998. Different lobes have been considered and different thicknesses of shielding can approximate different situations such as helmet, spacesuit, and various possible shielding depths within a habitat. Such determined pivot energy, the Pcc of the fitting, and the fitted parameters of the linear function for each case are given in Table 2. These values can be used as a look-up table to forecast or nowcast the dose deposit inside a human head in space (under different shielding depths as well as in various parts of the brain) based solely on the SEP events fluence at these pivot energies.

**Table 2:** The fitted parameters of Eq. (2) for deriving the SEP events induced dose $D_{\mathrm{{Brain}}}$ at a certain part of the brain or the head under different thickness of Al shielding. The determined pivot energy $I_{\mathrm{{Pivot~energy}}}$ of the SEP events for different cases is also given. The fitted parameter *a* has the unit of cGy $\cdot$ sr  $\mathrm {cm}^{2}$ MeV and *b* with unit of cGy.

	Scenarios	Pivot energy (MeV)	a$\times 10^{6}$	b	$\hbox {R}^2$
Human head	No shield	20	2.42 ± 0.04	15.69 ± 8.60	0.985
	2 mm Al	40	5.89 ± 0.03	2.048 ± 1.05	0.998
	5 mm Al	63	14.5 ± 0.08	0.721 ± 0.44	0.998
	2 cm Al	131	45.3 ± 0.25	0.114 ± 0.10	0.998
	5 cm Al	208	70.6 ± 0.33	0.021 ± 0.03	0.999
	10 cm Al	300	77.6 ± 0.46	0.011 ± 0.01	0.998
Lobes of brain protected by 2 mm of shield	Frontal	75	18.0 ± 0.13	0.57 ± 0.36	0.997
	Parietal	90	28.4 ± 0.23	0.40 ± 0.33	0.996
	Temporal	90	27.4 ± 0.20	0.38 ± 0.27	0.997
	Occipital	90	28.3 ± 0.18	0.38 ± 0.26	0.997
	Hippocampus	120	26.8 ± 0.35	0.18 ± 0.11	0.990

## Summary and conclusions

Among various effects that may cause health problems for astronauts on long-duration missions to outer space such as the Moon or Mars, space radiation deserves special attention because it has known long-term effects, but can also become critical in the course of short time periods. Effects on the CNS are important because they can lead to changes in motor function and behavior or neurological disorders, which can have devastating effects during a deep space mission.

The radiation induced by the background GCRs is omnipresent but mostly stable and easier to predict. However, the radiation associated with SEP events is sporadic, highly variable from events to events, and may cause severe acute syndromes to astronauts if no sufficient protection is prepared, such as during extravehicular activities. This requires the most straightforward and easy-to-apply approach to forecast the SEP events induced radiation effect during the course of a manned mission.

In the previous work^41^, we have established a set of GEANT4 Monte Carlo simulations to describe the interactions of highly energetic particles with an actual human head structure and lobes inside the brain. The dose deposit in different lobes of the brain, in the hippocampus region and in the whole human head under different shielding scenarios, has been summarized as statistical functions, which depend on the primary particle energy of the SEP events/GCRs. In this paper, we apply these functions to the GCRs and SEP events environment that an astronaut may encounter in deep space.

First, we fold these BRFs with Badwhar O’Neil^50^ input spectra for different GCR modulation parameters and derive the doses in different lobes, and in the hippocampus region. The overall dose rate is between 99 and 437 $\upmu$Gy/day for solar maximum and minimum conditions. Not unexpectedly, there is no substantial difference between the dose rate in different lobes for GCRs. Increased shielding thickness, however, shows a noticeable reduction in the dose rate experienced by the brain. Reductions are 12% and 25% for 2 mm and 10 cm of Al shielding during solar maximum, respectively, but reach to 14% and 37% during solar minimum conditions, respectively.

This sensitivity to shielding is even more pronounced for SEP events because they typically cover a lower energy range than GCRs. Shielding by 2 mm of Al can reduce the dose from SEP events by more than 65%, an increased shielding by 2 cm of Al reduces it by 90%. This reduction is sufficient to protect astronauts against acute radiation syndromes for all SEP events encountered during the space age.

Although the differences in dose experienced by the different lobes during SEP events are small, the frontal lobe sees the largest dose because it has the largest area close to the surface of the human brain. On the other hand, the hippocampus region, which is located deep inside the temporal lobe absorbed a lower dose than the outer parts. The low-energy part of SEP events deposits most of its energy in the outer surface of the brain, thus mostly affecting the frontal lobe. This could be mitigated by a clever helmet design.

The dose-fluence relationships are shown for proton in different energies for each scenario to determine the best correlation coefficient, and then, the pivot energy. Finally, we formulate the correlation between SEP events intensity and the brain dose for each scenario that have been reported in Table 2. Using this function and parameter for each scenario, one can quickly calculate the SEP events induced dose at a certain part of the brain or the human head considering the different thickness of shielding around, given the intensity of the SEP events at the pivot energy. These results show that the pivot energy in the a human head with 10 cm of Al as a shield is about 300 MeV, which is the same as the pivot energy on the surface of Mars with $\sim$ 22 g/$\hbox {cm}^{2}$ of vertical column depth of $\hbox {CO}_{{2}}$^39^.

The statistical analysis of the resulted dose versus the properties of the incoming SEP events spectra of 50+ historical events provides us some empirical correlations which can be directly applied to predict the dose in the brain from the initial SEP-event fluence, thus minimizing the complication of the forecast while keeping the accuracy as a full Monte Carlo simulation and saving the computational power. We note, however, that using only historical event data bears with it the risk that one is not prepared for an event which was not known in the past. Here we rely on our knowledge which has been accumulated in the space age and is hopefully short compared with the many generations of humans that will visit space in the future.

The pivot energies for the head, various lobes and the hippocampus region, range between about 20 and 300 MeV, mainly depending on different shielding depths. We suggest the importance of in-situ (outside the vessel or the base) measurement of particle fluences at these energies, which are an important parameter for a direct prediction of SEP radiation impacts on human brains.

The radiation effect on the hippocampus of mice, which is an important structure for the formation of long-term episodic memory has been studied in^55^. Their results show that 1 Gy of proton radiation can produce long-term changes to neuronal electrophysiological states. With 0.1 Gy radiation from incident protons, object recognition memory is impaired three months following the irradiation experiment^56^. As shown in Table 1, our statistical analysis includes multiple cases with the event radiation dose exceeding 0.1 Gy, even under 2 or 5 mm of shielding. Of course, the human head and brain structure is different from that of a mouse, and it is not certain that the same neurological impact can be expected. However, together with these biological experiments, our statistical study suggests that the drastically-enhanced dose induced by SEP events may pose long-term effects to the CNS of brains in space and even lead to their malfunction in a long term. This should be taken into consideration for long-duration space missions, such as future missions to Mars.
